# Detection of Incidental Esophageal Cancers on Chest CT by Deep Learning

**DOI:** 10.3389/fonc.2021.700210

**Published:** 2021-09-16

**Authors:** He Sui, Ruhang Ma, Lin Liu, Yaozong Gao, Wenhai Zhang, Zhanhao Mo

**Affiliations:** ^1^China-Japan Union Hospital of Jilin University, Changchun, China; ^2^Radiology Department, Weifang People’s Hospital, Weifang, China; ^3^Shanghai United Imaging Medical Technology Co., Ltd., Shanghai, China

**Keywords:** deep learning, convolutional neural network, chest CT, esophageal cancer, v-net

## Abstract

**Objective:**

To develop a deep learning-based model using esophageal thickness to detect esophageal cancer from unenhanced chest CT images.

**Methods:**

We retrospectively identified 141 patients with esophageal cancer and 273 patients negative for esophageal cancer (at the time of imaging) for model training. Unenhanced chest CT images were collected and used to build a convolutional neural network (CNN) model for diagnosing esophageal cancer. The CNN is a VB-Net segmentation network that segments the esophagus and automatically quantifies the thickness of the esophageal wall and detect positions of esophageal lesions. To validate this model, 52 false negatives and 48 normal cases were collected further as the second dataset. The average performance of three radiologists and that of the same radiologists aided by the model were compared.

**Results:**

The sensitivity and specificity of the esophageal cancer detection model were 88.8% and 90.9%, respectively, for the validation dataset set. Of the 52 missed esophageal cancer cases and the 48 normal cases, the sensitivity, specificity, and accuracy of the deep learning esophageal cancer detection model were 69%, 61%, and 65%, respectively. The independent results of the radiologists had a sensitivity of 25%, 31%, and 27%; specificity of 78%, 75%, and 75%; and accuracy of 53%, 54%, and 53%. With the aid of the model, the results of the radiologists were improved to a sensitivity of 77%, 81%, and 75%; specificity of 75%, 74%, and 74%; and accuracy of 76%, 77%, and 75%, respectively.

**Conclusions:**

Deep learning-based model can effectively detect esophageal cancer in unenhanced chest CT scans to improve the incidental detection of esophageal cancer.

## Highlights

1. The deep learning-based model can help radiologists reduce missed diagnosis of esophageal cancer on unenhanced chest CT images.

2. Besides recognizing esophageal cancer cases, VB-Net is also able to localize the thickening / carcinogenesis positions in CT images of esophageal cancer positive patients.

## 1 Introduction

Esophageal cancer, originating from the esophageal mucosa, is one of the most common malignant tumors in the world (1). Smoking is recognized as the most common risk factor for esophageal cancer (2). The mortality rate of esophageal cancer ranks sixth worldwide (3, 4), mainly due to its late diagnosis (5), rapid development, and fatal prognosis in most cases (6). Additionally, there is an increasing trend in the incidence rate of esophageal cancer in the recent years (7–9). Despite improvements to the management and treatment of esophageal cancer, the 5-year survival rates (~10%) and 5-year post esophagectomy survival rates (15%–40%) are still extremely poor (10). Advances in the early detection and treatment of esophageal cancer have greatly contributed to improving the survival rates over the past several years (11), so it is clear that an early detection is of great benefit. In the current diagnosis and treatment process, the screening and diagnosis of esophageal cancer still require endoscopy and biopsy. However, these procedures are costly, invasive, prone to sampling errors (12), plus, there is a lack of professionally trained endoscopists (13). Through a previous research, it has been shown that thickening of the esophageal wall is a key manifestation of esophageal cancer (14, 15). As a widely used examination method, CT imaging can be used to help detect esophageal cancers (16). Radiologists use the abnormal thickening of the esophageal wall as the diagnostic basis to indicate the occurrence of esophageal cancer, thereby prompting the patient to further endoscopy to verify the diagnosis. However, radiologists rely on the provision of the medical history, and the reading ability is limited by the low resolution of soft tissue in CT. These factors lead to a high false negative rate of esophageal cancer in the day-to-day practice.

Artificial intelligence (AI), especially deep learning, has emerged as a promising field in radiology and medicine. AI has already been used to perform tasks such as detecting pulmonary nodules (17), staging liver fibrosis (18), classifying pulmonary artery-vein (19), segmenting liver tumor automatically (20), and detecting bone fracture (21), hemorrhage, mass effect, and hydrocephalus (HMH) (22). There have also been several reports on its application in esophageal lesion diagnosis using endoscopy (23–25). However, endoscopy is an invasive examination and is not commonly used. In this article, we propose to detect esophageal lesions in chest CT using deep learning, which is truly cutting-edge and non-invasive. With the popularity of chest CT scanning and reductions in the radiation dosage, AI is becoming increasingly useful as a tool to improve the performance of the detection of incidental esophageal cancer.

## 2 Materials and Methods

### 2.1 Data Preparation

#### 2.1.1 Data Set Used to Construct the Deep Learning-Based Model (Data Set 1)

All the procedures were in accordance with the ethical standards of the ethics committee on human experimentation of our hospital. We retrospectively collected 141 patients (mean age: 57.4, age range: 34 to 87) with esophageal cancer and 273 negative cancer patients (mean age: 41.7, age range: 18 to 73) who underwent unenhanced chest CT from February 2017 to April 2019 in the China-Japan Union Hospital of Jilin University. This was called Data set 1. Then, Data set 1 was randomly divided into a training set and a validation set using the ratio of training set to validation set 7:3 **(****Tables 1**, **2****)**. The reasons for the CT scans are that chest CT should be done routinely before hospitalization in our hospital or screening for pulmonary nodules.

**Table 1:** Patient characteristics in the data set 1 and data set 2.

			Data set 1				Data set 2
			Training (N = 99)		Validation (N = 42)		
Age (yr, mean ± sd)			60.15 ± 8.61		50.76 ± 11.44		50.83 ± 9.72
Sex							
male			94		40		45
female			5		2		7
Lesion location(N=)							
Ut			9		3		5
Mt			65		26		35
Lt			25		13		12
T stage(N=)							
T1			18		9		24
T2			32		9		14
T3			43		19		9
T4			6		5		0
Tumor size, cm							
mean ± sd			1.27 ± 0.40		1.53 ± 0.61		0.87 ± 0.12
range			0.6–2.2		0.6–3.2		0.6–1.2
squamous cell carcinoma			90		40		46
adenocarcinoma			9		2		6

Ut, upper thoracic esophagus; Mt, middle thoracic esophagus; Lt, lower thoracic esophagus.

mean ± SD means mean ± standard deviation, N means quantity.

**Table 2:** Non-esophageal cancer subject characteristics in the data set 1 and data set 2.

	Data set 1		Data set 2
	Training (N = 223)	Validation (N = 50)	
Age (yr, mean ± sd)	51.20 ± 10.71	43.00 ± 12.93	42.08 ± 12.65
Sex			
male	98	34	36
female	125	16	12

mean ± SD means mean ± standard deviation, N means quantity.

Inclusion criteria: Data set 1, patients with esophageal cancer were selected based on the availability of a chest CT scan before surgery, surgical pathology or endoscopic pathology confirming esophageal cancer, and patients had no other disease that could cause thickening of the esophageal wall. For the negative cancer subjects, patients needed a chest CT scan and be negative for esophageal cancer in the following two years.

Exclusion criteria: Patients were excluded from the data set if any of the clinical data was incomplete or the chest CT scans taken were of poor quality.

#### 2.1.2 Data Set Used for the Clinical Evaluation of the Deep Learning-Based Model (Data Set 2)

In order to evaluate the clinical performance of this deep learning-based model, we collected 48 normal cases and 52 cases of esophageal cancers that were missed by all radiologists in the hospital but confirmed by pathology from January 2017 to December 2019. This was named as Data set 2. **(****Tables 1**, **2****)** For the 48 normal cases, some of these patients underwent chest CT because of chest pain, progressive dysphagia, screening for pulmonary nodules, and some patients need a routine chest CT examination before hospitalization. In addition to the inclusion criteria mentioned in Data set 1, the set also includes cases that have been pathologically confirmed but missed by the radiologist with the same exclusion criteria as above.

#### 2.1.3 Computed Tomography (CT) Image Acquisition

All images were scanned by the Toshiba Medical Systems CT scanner (Tochigi, Japan), Siemens Healthcare CT scanner (Munich, Germany) and GE Healthcare CT scanner (Waukesha, WI) with a section thickness of 5 mm and an image slice matrix of 512 × 512 at China-Japan Union Hospital. Automatic tube current modulation techniques were adopted with the tube voltage set at 120 kVp. All images were available for review in our PACS (RISGC 3.1.S18.2, Carestream Health, Inc.).

### 2.2 Deep Learning-Based Esophageal Cancer Detection

#### 2.2.1 V-Net for Esophagus Segmentation and Thick Esophagus Wall Localization

**Figure 1:**
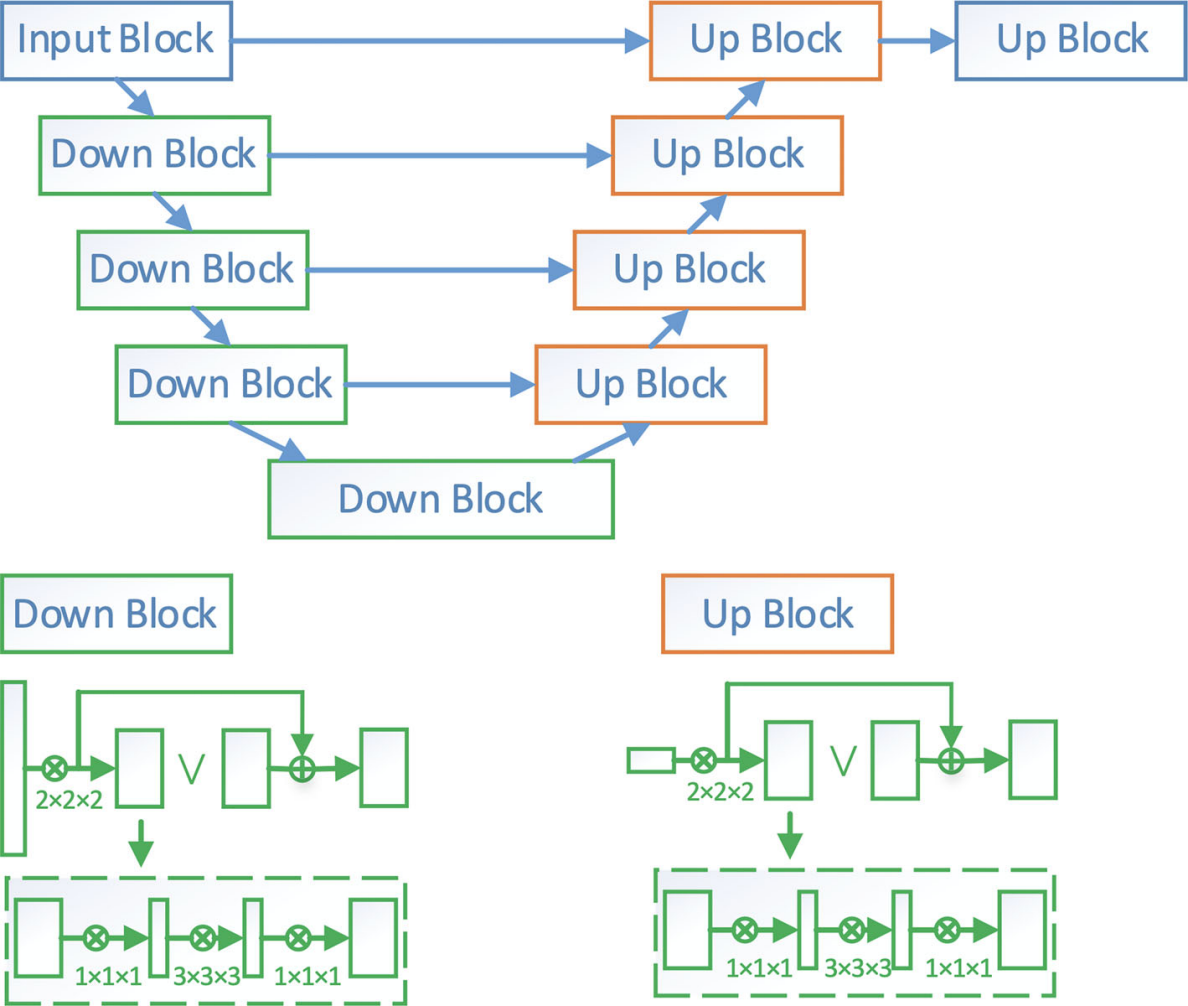
The network architecture of VB-Net. Down block is a down-sampling network block. Its detailed architecture is shown in the left-bottom corner of the figure, where each rectangle is a convolutional layer. Up block is an up-sampling network block. Its details are shown in the right-bottom corner of the figure.

V-Net is a widely adopted deep learning network for 3D volumetric segmentation. In this paper, we adopt a modified V-Net architecture named VB-Net to segment the esophagus from the CT images. The network architecture is shown in the **Figure 1**. It consists of two paths, a contracting path for extracting the global image context and an expanding path for incorporating the low-level detail information. By combining the high-level and low-level information, VB-Net is able to accurately capture the boundary of esophagus.

**Figure 2:**
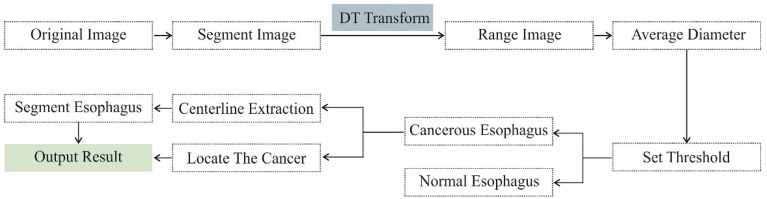
Deep learning-based model formation process.

After segmentation of the esophagus, the average boundary distance of points within the esophagus can be computed *via* a distance transform. An optimal threshold is determined based on the cross-validation to discriminate between esophageal cancer and normal patients **(****Figures 1**, **2****)**.

Besides recognizing the esophageal cancer cases, the algorithm is also able to localize the thickening/carcinogenesis position in the CT images of patients with cancer. To exclude the physiological thickening near the cardia and esophageal entrance (tracheal bifurcation), 2 cm away from both the beginning and end of the esophagus is excluded for thickening detection. Similarly, if there is more air in the esophagus, the diameter of the esophagus would also be larger, which could confuse the AI algorithm to recognize it as a thickening cancer position. To filter out such cases, the air component is extracted from the esophagus using a threshold (i.e., HU value < -900), esophageal CT slices with more than 20% air occupation are filtered out. Finally, slices with the maximum diameters are picked as thickening/carcinogenesis position.

To further divide the esophagus into the upper/middle/lower sections, the centerline of the esophagus is first extracted from the binary segmentation using the thinning algorithm provided in the OpenCV C++ library. Then, the centerline is divided into three sections using the ratio 1:1:3.

#### 2.2.2 Evaluation of Deep Learning-Based Model

To evaluate the effectiveness of deep learning in recognizing esophageal cancer patients, the receiver operating characteristic (ROC) curve, accuracy, sensitivity, and specificity are used as the reporting metrics. The ROC curve can analyze the classification performance of the model independently of the average diameter threshold. For the accuracy, sensitivity, and specificity, an optimal threshold is first determined based on a cross validation, and then, their values are calculated and reported on both the validation and testing datasets.

### 2.3 Clinical Evaluation of the Deep Learning-Based Model

To explore the benefits of using a deep learning model for esophageal cancer detection, a comparison experiment was performed. The control group of three radiologists without the assistance of the model would be compared to the model by itself and the same group of radiologists with the assistance of the model. The experiment was conducted as follows.

The deep learning-based model independently processed the CT images of Data set 2 and marked its candidate esophageal cancers area with green boxes. Three radiologists (with 5**–**7 years of CT diagnostic experience) independently read the CT images of Data set 2 without instructions to specifically look for esophageal cancer to mimic the daily diagnostic process of the radiologists. The radiologists then described the esophageal cancers they diagnosed in the CT reports.

After the 30-day memory washout period, the same three radiologists read the CT images of Data set 2 with the assistance of the deep learning-based model without instructions to specifically look for esophageal cancer and described esophageal cancers they diagnosed in the CT reports.

We separately compared the results of these three modes with pathology to determine whether they diagnosed the esophageal cancers correctly. There was a correlation between the esophageal cancer location and the endoscopy or pathology report. False positive and negative diagnosis results were also recorded. Then, the sensitivity, specificity, and accuracy of the three reading modes were calculated.

### 2.4 Statistical Analysis

The performance of the three different reading modes above was compared by using Student’s t-test. All the statistical analyses were performed by using a software (SAS version 9.4; SAS Institute, Cary, NC). The significance level or P-value threshold was set to 0.05.

## 3 Results

### 3.1 The Training Loss (Dice Loss) for the Segmentation Model

**Figure 3:**
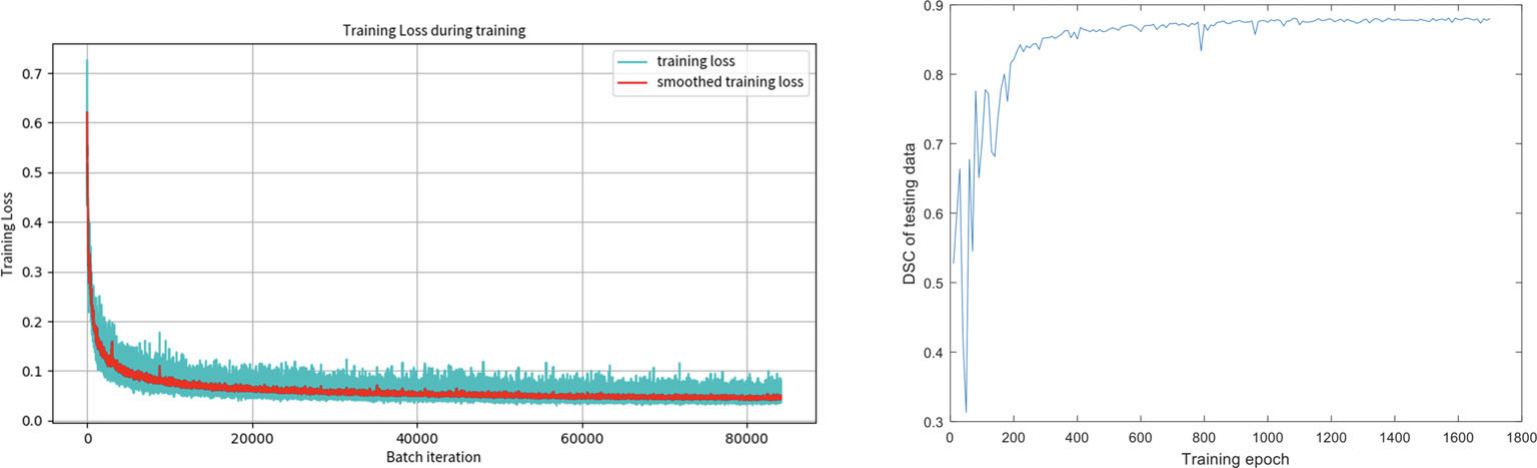
The training loss (Dice loss) for the segmentation model and the Dice coefficients of the segmentation models at different training epochs on our test data set.

**Figure 3** shows the training loss (Dice loss) for the segmentation model. The Dice similarity coefficients (DSC) of the segmentation models at different training epochs on our test data set were also evaluated, shown in the second figure of **Figure 3**. It can be seen that DSC keeps increasing at the beginning of the training and stabilizes after 400 epochs, which suggests that the training process has converged.

### 3.2 The Performance of the Deep Learning-Based Model

**Figure 4:**
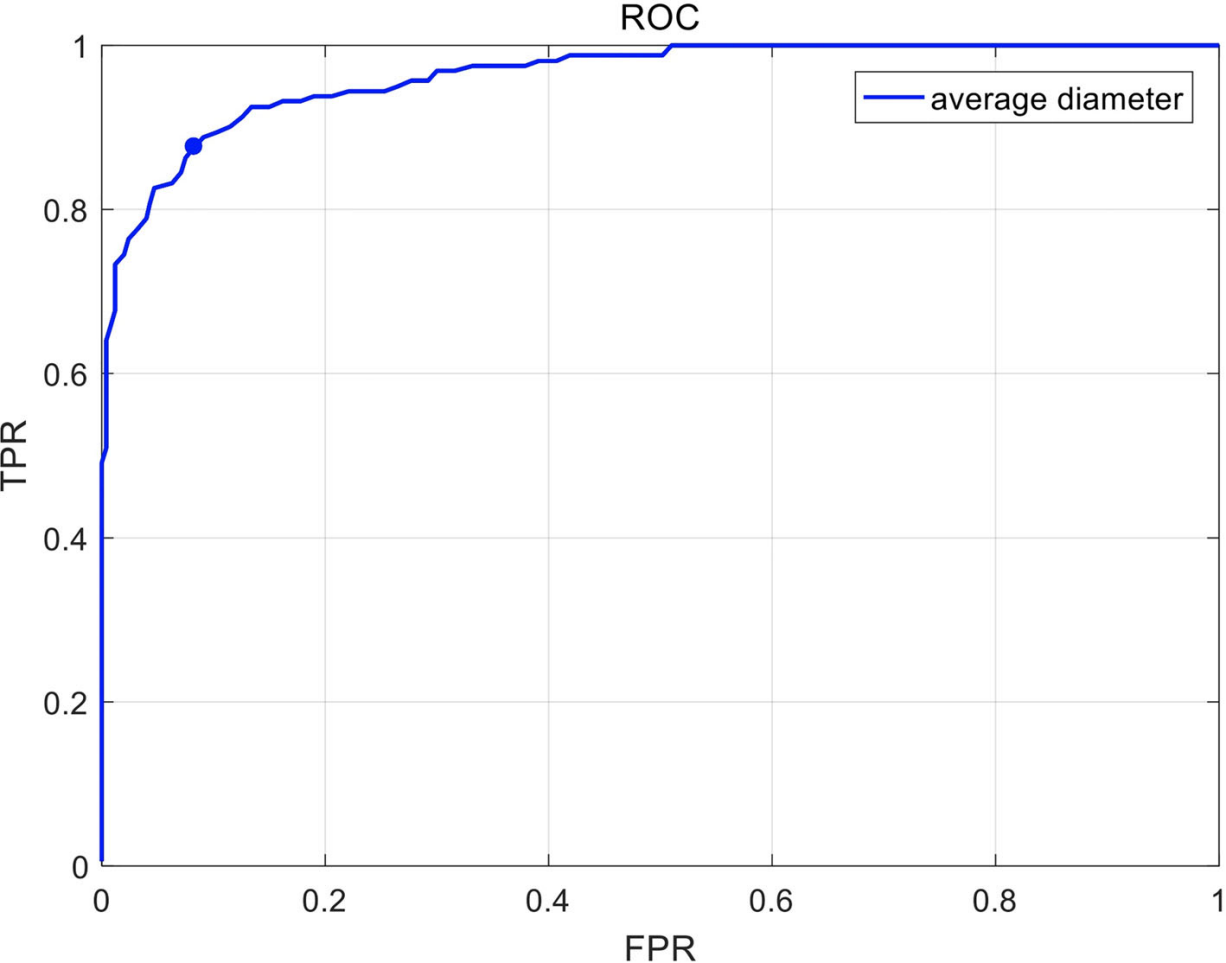
The receiver operating characteristic (ROC) curve in recognizing esophageal cancer patients on unenhanced chest CT scans. Sensitivity and specificity in the figure are calculated by varying the threshold of average diameter.

The construction of the deep learning-based model used 414 CT images (141 esophageal cancer cases and 273 normal cases). The ROC curve shown in **Figure 4** was computed by varying the threshold of the average diameter. The deep learning-based model yielded an AUC (Area under Curve) of 0.96. The sensitivity and specificity of the deep learning-based model are 88.8% and 90.9% on the randomly splitted validation set, respectively.

### 3.3 Detection of the 48 Normal Esophagus and 52 Missed Esophageal Cancers (Date Set 2) by the Deep Learning-Based Model

**Figure 5:**
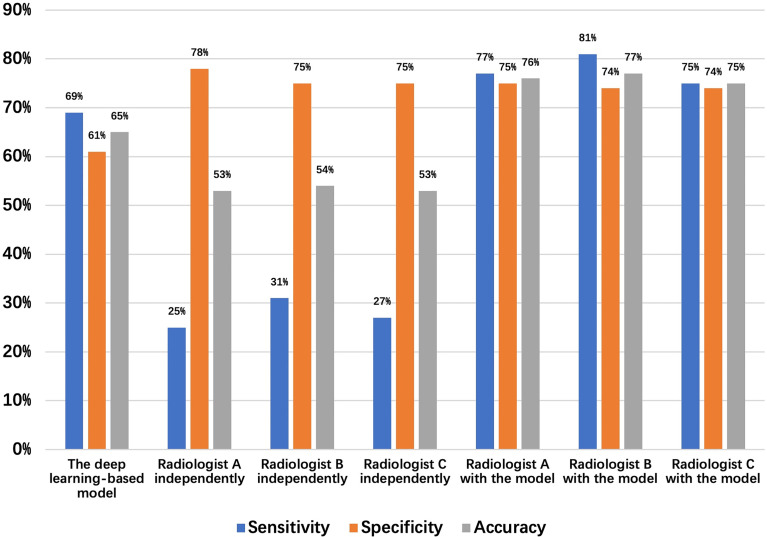
Detection results of the 48 normal esophagus and 52 missed esophageal cancers (date set 2) by the deep learning-based model and radiologists.

A total of 64 sites were detected by the deep learning-based model, including 8 in the Ut (Upper thoracic esophagus), 37 in Mt (Middle thoracic esophagus), and 19 in Lt (Lower thoracic esophagus). Taking into account that this model may detect more than one abnormality in the same patient, true positives, false positives, true negatives, and false negatives are 36 (Ut2, Mt25, Lt9), 28 (Ut6, Mt12, Lt 10), 44 (Ut7, Mt26, Lt 11), and 16 (Ut3, Mt10, Lt3), respectively. The sensitivity, specificity, and accuracy of the deep learning-based model are 69%, 61%, and 65%, respectively, as shown in **Figure 5**. Among these esophageal cancer cases detected correctly, the detection rate of Mt esophageal cases is higher than that of the Ut and Lt locations **(****Tables 3**, **4****)**.

**Table 3:** The outcomes detected by the deep learning-based model, the three radiologists independently, and with the assistance of the deep learning-based model.

			Candidate cancers	True positives	False positives	True negatives	False negatives
			Ut/Mt/Lt	Ut/Mt/Lt	Ut/Mt/Lt	Ut/Mt/Lt	Ut/Mt/Lt
The deep learning-based model			8/37/19	2/25/9	6/12/10	7/26/11	3/10/3
Radiologist A independently			5/18/3	2/8/3	3/10/0	10/24/12	3/27/9
Radiologist B independently			7/19/5	3/10/3	4/9/2	13/21/11	2/25/9
Radiologist C independently			7/13/9	4/9/1	3/4/8	11/25/10	1/26/11
Radiologist independently(avg.)			6.3/16.7/5.7	3/9/2.3	3.3/7.7/3.3	11.3/23.4/11	2/26/9.7
Radiologist A with the model			11/32/13	5/29/6	6/3/7	13/21/13	0/6/6
Radiologist B with the model			10/35/13	4/31/7	6/4/6	12/21/13	1/4/5
Radiologist C with the model			12/30/13	4/28/7	8/2/6	14/23/9	1/7/5
Radiologist with the model(avg.)			11/32.3/13	4.3/29.3/6.7	6.7/3/6.3	13/21.7/11.6	0.7/5.7/5.3
P Value (DM *vs* RI)			0.002	0.002	0.002	0.038	0.002
P Value (DM *vs* RM)			0.002	0.000	0.130	0.184	0.000
P Value (RI *vs* RM)			0.013	0.039	0.001	0.020	0.039

Avg. means average. Ut, upper thoracic esophagus; Mt, middle thoracic esophagus; Lt, means lower thoracic esophagus, DM, The deep learning-based model; RI, Radiologist independently; RM, Radiologist with the model.

**Table 4:** The sensitivity, specificity, and accuracy in diagnosing by the deep learning-based model and radiologist with or without the assistance of the deep learning-based model.

			Sensitivity		Specificity		Accuracy		
			Ut/Mt/Lt		Ut/Mt/Lt		Ut/Mt/Lt		
The deep learning-based model			40%/71%/75%		54%/68%/52%		50%/75%/61%	
Radiologist A independently			40%/23%/25%		77%/71%/100%		68%/46%/63%	
Radiologist B independently			60%/29%/25%		76%/70%/85%		73%/48%/56%	
Radiologist C independently			80%/26%/8%		79%/86%/89%		79%/53%/37%	
Radiologist independently(avg.)			60%/26%/19%		77%/75%/77%		73%/49%/51%	
Radiologist A with the model			100%/83%/50%		68%/88%/65%		75%/85%/59%	
Radiologist B with the model			80%/89%/58%		67%/84%/68%		70%/87%/65%	
Radiologist C with the model			80%/80%/58%		64%/92%/60%		67%/85%/59%	
Radiologist with the model(avg.)			86%/84%/56%		66%/88%/65%		70%/85%/61%	

Avg. means average.

Three radiologists (A, B, and C with 5–7 years of CT diagnostic experience) were enrolled in this comparative study. The average numbers of candidate esophageal cancer sites, true positives, false positives, true negatives, and false negatives detected by the three radiologists are 28.7 (Ut6.3, Mt16.7, Lt5.7), 14.3 (Ut3, Mt9, Lt2.3), 14.3 (Ut3.3, Mt7.7, Lt3.3), 45.7 (Ut11.3, Mt23.4, Lt11), and 37.7 (Ut2, Mt26, Lt9.7), respectively. Thus, the mean sensitivity, specificity, and accuracy are 27.5%, 76.2%, and 53.6%, respectively **(****Tables 3**, **4****)**.

After the 30-day memory washout period, with the assistance of the model, the same three radiologists read Data set 2 again. This time, they are aided by the model and can add or rule out esophageal cancer suggested by the model before making their final diagnosis.

The same three radiologists marked 56 cases of candidate esophageal cancer, respectively, 56 (Ut11, Mt32, Lt13),58 (Ut10, Mt35, Lt13), and 55 (Ut12, Mt30, Lt13). Their specificities are 75%, 74%, and 74%, and corresponding accuracies are 76%, 77%, and 75%. The average numbers of the candidate esophageal cancer cases, true positives, false positives, true negatives, and false negatives by the three radiologists are 56.3 (Ut11, Mt32.3, Lt13), 40.3 (Ut4.3, Mt29.3, Lt6.7), 16 (Ut6.7, Mt3, Lt6.3), 46.3 (Ut13, Mt21.7, Lt11.6), and 11.7 (Ut0.7, Mt5.7, Lt5.3), respectively. Thus, the mean sensitivity, specificity, and accuracy are 77.5%, 74.3%, and 75.8%, respectively **(****Tables 3**, **4** and **Figure 5****)**.

As can be seen in **Table 3**, we found that the total number of cancer cases detected correctly by the radiologists with the assistance of the deep learning-based model was more than those detected by the deep learning-based model alone or radiologists without the assistance of the deep learning-based model.

**Figure 5** shows the sensitivity, specificity, and accuracy of the diagnosis by the deep learning-based model and the three radiologists with or without the assistance of the deep learning-based model, the best performance is achieved by the radiologists with the assistance of the deep learning-based model.

### 3.4 Causes for True/False Positives and True/False Negatives in the Preoperative Computed Tomography (CT) Scan

The number of candidate cancer cases marked by the deep learning-based model was 64. The number of false positives was 28, and the number of false negatives was 16.

**Figure 6:**
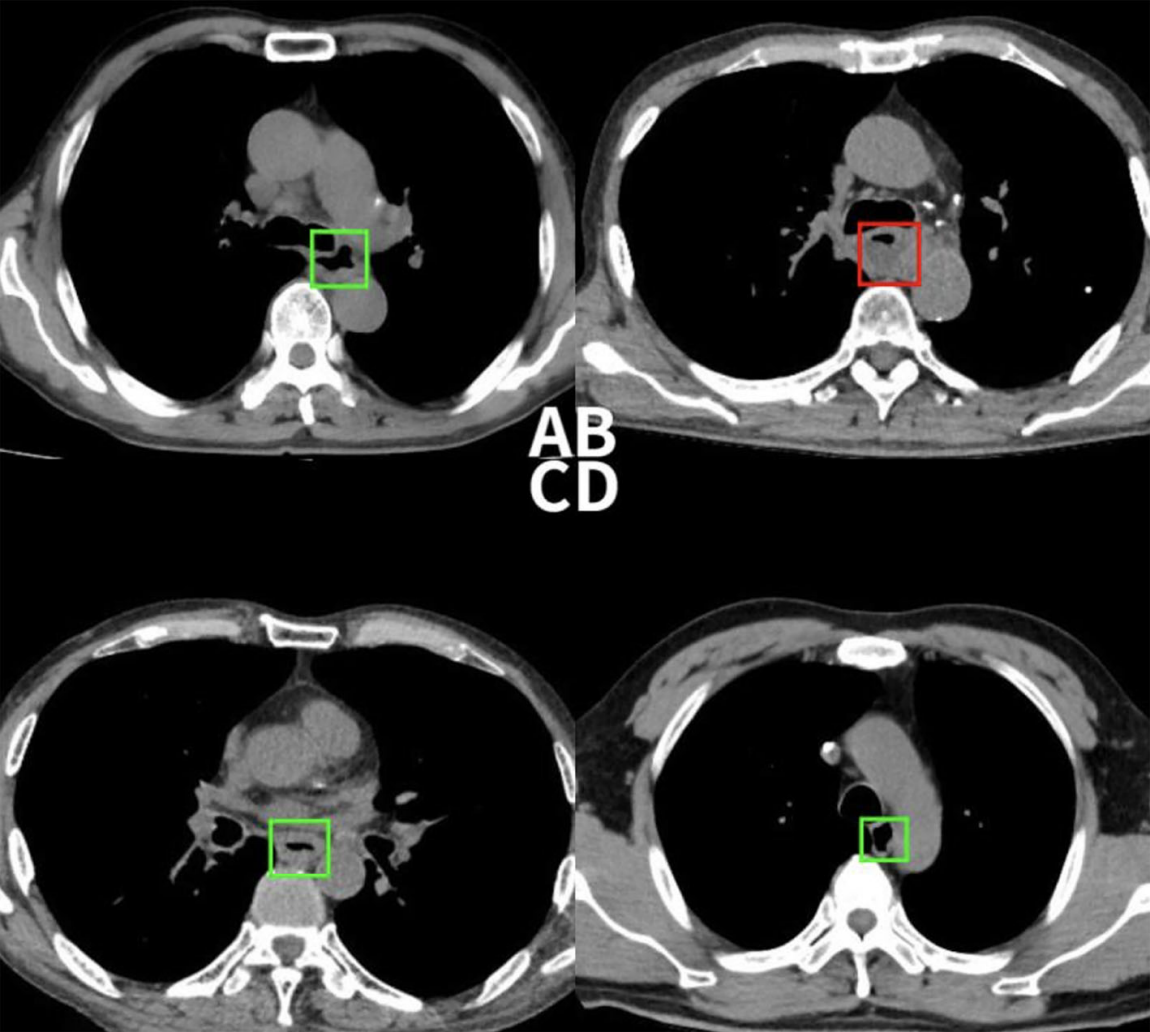
**(A)** This case was detected correctly by the deep learning-based model, but the radiologist missed. **(B)** This non-esophageal cancer subject was misdiagnosed as positive by the model due to the abnormal filling of the esophageal cavity. **(C)** This case was confirmed pathologically as esophageal inflammation, and the model mistaken it for esophageal cancer. **(D)** This case was successfully detected by the radiologist, but the model missed the diagnosis.

1. Causes for True positive: The direct imaging sign of esophageal cancer is the thickening of the esophageal wall, and our goal was to detect the abnormality of esophageal wall thickness, so as to esophageal cancer. Based on the experimental results, we can detect 88.8% of esophageal cancer cases correctly. With the aid of the model, radiologists have greatly improved the sensitivity of the diagnosis of esophageal cancer. (**Figure 5**, **6A**).

2. Causes for False positive: Among the 28 false positive cases, 11 were esophageal inflammation that caused esophageal mucosal edema, 14 were esophageal leiomyomas, and 3 were esophageal varices that caused the uneven thickening of the esophageal wall. Although the direct imaging sign of esophageal cancer is an abnormal thickening of the esophageal wall, thickening of the esophageal wall does not purely indicate esophageal cancer; inflammation, leiomyoma, etc. can also cause a thickening of the tube wall. In addition, due to the uncontrollable filling of the esophageal cavity in the chest CT, the detection sensitivity of patients with abnormal luminal filling could lead to the occurrence of false positives (**Figures 6B, C****)**.

3. Causes for True negative: The detection threshold was properly set to eliminate the interference caused by peristaltic rush and other changes in the thickness of the esophageal wall to a certain extent and will not over-prompt the changes in the thickness of the esophageal wall. Thus, it reduces the workload of radiologists for a second read as much as possible.

4. Causes for False negative: A total of 16 false negative cases were due to their small tumor sizes (carcinoma *in situ*). Because the model only focuses on detecting the thickness of the esophageal wall, other changes, e.g., texture, in the tube wall cannot be identified, resulting in smaller lesions being more difficult to be detected. Moreover, the deep-learning model cannot effectively extract indirect signs such as the blurring of the fat space around the tube wall, enlarged lymph nodes, and abnormal expansion of the lumen above the lesion, etc. also leading to false negatives (**Figure 6D**). As for now, the comprehensive observation of radiologists is still supplemental to the model detection.

Therefore, the complementary advantages of deep learning and radiologists can improve the efficacy of esophageal cancer detection.

## 4 Discussion

In the retrospective analysis, the majority of esophageal cancers can be identified from the CT images. The causes of failure to detect those cancer cases can be either missing the lesion or dismissing the lesion as a physiological thickening. The former is considered as a detection error, while the latter is an interpretation error that typically occurs when the morphologic structure of the abnormality is similar to normal ones in appearance. We aim to provide a model that can objectively and accurately identify esophageal abnormalities, thereby reducing a missed diagnosis. Based on this, we only selected pathologically confirmed cases of esophageal cancer for the model construction.

Massive endoscopic screening has a satisfactory performance on the detection of early esophageal cancers (26, 27). However, patients with esophageal cancers often have no obvious symptoms (28) in the early stage and are not commonly recommended for endoscopy. In addition, some patients are afraid of going through the procedure and may opt out. Massive endoscopic screenings of esophageal cancers are only carried out in areas with a high incidence of esophageal cancers. As the living standards of people rise and health awareness increases, chest CT is also becoming a routine health screening option. In view of the strong subjectivity of radiologists in the diagnosis of CT images, our model based on deep learning is highly objective in detecting esophageal cancer and can provide a higher degree of reliability in detecting abnormalities.

We adopted VB-Net in this study for two reasons. First, this model has been validated over thousands of CT images and in many organ segmentation problems (29, 30). It showed very promising results in tasks involving segmentation. Second, compared with the popular U-Net model, VB-Net is specifically designed for industry production purpose. It utilizes the bottle-neck structure to reduce the model size while keeping a similar segmentation accuracy. VB-Net model takes 11.1 MB while the U-Net model takes 459 MB. The small size of the model not only makes it easy to deploy, but also makes the runtime inference faster. In the task of CT esophagus segmentation, we conducted experiments that compared the segmentation accuracy of VB-Net with U-Net. **Table 5** shows the quantitative comparison. It can be seen that VB-Net achieved a slightly better segmentation accuracy than U-Net in terms of Dice coefficient and Hausdorff distance. More importantly, the major improvement of VB-Net over U-Net is the faster segmentation time (improved by nearly 10 times) and smaller model size (reduced by 41 times). These advantages make VB-Net preferable in AI product development.

**Table 5:** The quantitative comparison between VB-Net and U-Net.

	U-Net	VB-Net (chosen model)
Loss function	Dice loss	Dice loss
Model size	459 MB	11.1 MB
Segmentation Time	4.24 seconds	0.39 second
Dice coefficients	0.874 ± 0.053	0.881 ± 0.057
Hausdorff distance	5.54 ± 7.41 mm	5.53 ± 6.39 mm

To improve the deep learning-based model performance, we segmented the lesion through VB-Net and extracted the three-dimensional (3D) tumor volume, which is more stable and representative than a 2D analysis. Additionally, we constructed the deep learning-based model with a large number of training samples and set a reasonable threshold to ensure the repeatability of the model and the stability of the results. At the same time, the model can catch abnormalities in CT images quickly, which improves the efficiency of image reading.

From Data set 2, the deep learning-based model detected 69.2% (36 of 52) of the esophageal cancers that were originally missed by radiologists. Most of the lesions are small in size, and there is no obvious change in the thickness of the local esophageal wall. The size of the lesion is a significant indicator of the detection rate of the lesion. Esophageal cancer often occurs in the middle esophagus (31, 32), which is also consistent with the outcomes of the deep learning-based model.While a high sensitivity for the deep learning based model in detecting cancers is necessary for it to be valuable, a higher sensitivity will also increase the rate of false-positive findings, because a high rate of false-positive findings requires the radiologists to spend an extra time and effort in the CT reading process, excluding findings that are not real cancers. The deep learning-based model missed 30.8% of the cancers, which can be identified by the reviewing radiologists. It is of great significations when considering whether the deep learning-based model might be used as either a primary reader or as a concurrent reader, or as a secondary reader. For the deep learning-based model, to be used as a primary or concurrent reader, an extremely high sensitivity is needed because using it may potentially alter the way the radiologist reviews the images. The radiologist should not be too dependent on using the model to catch smaller cancers and lesions. Among the 16 cases of esophageal cancer missed by the model, 10 cases were because the lesion was too small to cause a thickening of the esophageal wall. Therefore, the next step is to improve the model, extract the characteristic values of non-thickened esophageal cancer esophageal cancers, and increase the sample size to increase the detection rate of non-thickened esophageal cancers. In the false positive diagnosis, although the lesions detected by the deep learning-based model are not esophageal cancers, they were also esophageal diseases that caused the thickening of the esophagus, indicating that the model has clinical applications even outside specifically detecting esophageal cancers.

The health awareness of people has increased, and chest CT becomes popular (33). The proposed deep learning model aims to reduce missed diagnosis of esophageal cancer by radiologists in the daily chest CT diagnosis process. Because radiologists often pay more attention to lung diseases such as lung nodules in the routine reading, we instructed that “the radiologists read the CT images as in their normal practices” without specifically looking for esophageal cancer. Therefore, such experimental results can truly reflect the auxiliary diagnosis function of this deep learning model in the process of chest CT reading. As other organs such as thyroid, heart, breast (34), etc. should also be checked, the present model can be used as a supplemental tool for assisted esophageal cancer detection.

There are some limitations in the study. First, the study was based on a single center. Second, the deep learning-based model we developed only depends on the thickening of the esophageal wall and cannot recognize the texture and other radiomic features of the lesion. Therefore, radiologists cannot be adequately prompted when the lesion is small and the esophageal wall has not thickened enough. Other diseases that lead to esophageal wall thickening cannot be distinguished from esophageal cancer using our model. Third, because the model cannot explicitly detect indirect imaging signs such as the blurring of the surrounding fat gap and enlarged lymph nodes, the sensitivity is also impacted. We can see that physicians missed more cases of T1 stage through Data set 2, accounting for about 46% [24/(24 + 29+9)] of the dataset. This is partially due to the relatively few cases in the T1 stage in our training samples than other stages. More T1 stage data will make the model more stable and robust. Moreover, a low radiation dose unenhanced chest CT is often ordered for lung cancer screening for smokers, and the incidence of esophageal cancer is also higher for this particular demographic. Next, we will continue to collect more low-dose lung CT data to make the model more adaptable to different clinical settings. Finally, we only performed analysis on the missed cases. As abnormal imaging signs are not obvious, and, in daily practice, esophageal cancer is not very common, the purpose of the deep learning model is to highlight those patients with a possible abnormality. In the future, we expect to integrate more cases from different centers to validate its feasibility and scalability for clinical use.

## 5 Summary Statement

The deep learning-based model can assist radiologists in detecting esophageal cancer on chest CT to reduce the incidence of a missed diagnosis.

## Data Availability Statement

The original contributions presented in the study are included in the article/supplementary material. Further inquiries can be directed to the corresponding author.

## Ethics Statement

The studies involving human participants were reviewed and approved by The ethical standards of China-Japan Union Hospital of Jilin University ethics committee on human experimentation. Written informed consent to participate in this study was provided by the legal guardian/next of kin of the participants.

## Author Contributions

RM and HS are deeply involved in each stage of this work, including the methodology discussion, experiment design, and manuscript editing. All authors contributed to the article and approved the submitted version.

## Funding

This study was supported by the Jilin Province Science and Technology Development Plan Project (No.20200601007JC).

## Conflict of Interest

Authors YG and WZ where employed by Shanghai United Imaging Medical Technology Co., Ltd.

The remaining authors declare that the research was conducted in the absence of any commercial or financial relationships that could be construed as a potential conflict of interest.

## Publisher’s Note

All claims expressed in this article are solely those of the authors and do not necessarily represent those of their affiliated organizations, or those of the publisher, the editors and the reviewers. Any product that may be evaluated in this article, or claim that may be made by its manufacturer, is not guaranteed or endorsed by the publisher.

## Abbreviations

CNN, Convolutional neural network; Ut, Upper thoracic esophagus; Mt, Middle thoracic esophagus; Lt, Lower thoracic esophagus; ROC, Receiver operating characteristic; AUC, Are under curve; Al, Artificial intelligence; 3D, Three dimensional.
